# Psycho-demographic and clinical predictors of medication adherence in patients with bipolar I disorder in a university hospital in Egypt

**DOI:** 10.4102/sajpsychiatry.v26i0.1437

**Published:** 2020-02-10

**Authors:** Tarek A. Okasha, Doaa N. Radwan, Hussien Elkholy, Heba M.F.M. Hendawy, Eman M.M.E. Shourab, Ramy R.A. Teama, Ahmed S. Abdelgawad

**Affiliations:** 1 Department of Neurology and Psychiatry – Institute of Psychiatry, Faculty of Medicine, Ain Shams University, Cairo, Egypt; 2 Ministry of Health and Population, Cairo, Egypt

**Keywords:** bipolar disorder, adherence, medication, insight, illness, severity

## Abstract

**Background:**

Poor adherence to treatment is one of the main challenges to symptom control and preventing recurrence in bipolar disorder (BD). Numerous studies have established an association between patients’ poor adherence and an increased risk of recurrence, relapse of the symptoms and admission to hospital.

**Aim:**

To study the socio-demographic and clinical factors associated with medication nonadherence in patients with BD who were admitted to the hospital.

**Setting:**

The study was conducted at the Institute of Psychiatry, Ain Shams University.

**Methods:**

A 1-year longitudinal prospective study of 110 patients, aged 18–60 years, with BD-I. Young Mania Rating Scale, Clinical Global Impression, Global Assessment of Functioning, Sheehan Disability Scale and Insight and Treatment Attitude Questionnaire were applied before and 6 months after discharge. Adherence was measured using the Morisky 8-Item Medication Adherence Scale. Sociodemographic data and level of functioning were studied in relation to adherence.

**Results:**

Higher adherence was noticed in female, married and older patients and those with a higher level of education. However, low adherence was more common in male, non-married and less educated patients. Follow-up after 6 months revealed that the high adherence group scored the lowest in terms of disability. Meanwhile, the low adherence group scored the highest scores in disability.

**Conclusion:**

Several socio-demographic and clinical variables were found to be associated with a low adherence rate to the prescribed medication in patients with BD-I. Age and impaired insight were found to be significant predictive factors for non-adherence.

## Introduction

Bipolar disorder (BD) is one of the most severe psychiatric disorders that are estimated to have a prevalence of around 0.6% for type I and 0.4% for type II.^1^ It is one of the top 10 disorders causing disability worldwide,^2^ as it has been identified by the World Health Organization (WHO) as the sixth cause of years lost because of disability in young adults.^3^ Non-adherence is recognised as a big challenge particularly in the treatment of patients with chronic psychiatric disorders.^4^ Despite the plethora of evidence-based anti-bipolar medications, including lithium, anticonvulsant mood-stabilising medications and antipsychotics,^5,6^ the level of adherence to treatment amongst BD patients remains unsatisfactory. Many studies have reported that approximately half of patients with BD are poorly adherent to medications (non-adherence rate ranging from 20% to 70%).^7,8,9,10^ Moreover, experts estimate that commonly patients with BD take only 51% – 70% of their recommended doses.^11^

According to the WHO, adherence is defined as the extent to which a person’s behaviour-taking medication, following a diet, and/or executing lifestyle changes - corresponds with agreed recommendations from a health care provider.^12^ It was agreed that a patient is considered adherent if he or she takes 80% or more of his or her medication,^11^ although some studies have used a more conservative definition of missing 30% or more of prescribed medications.^13^ Patients who take 50% or more of medication are considered as partially adherent, whereas patients who do not take medication for a week or more are considered as non-adherent.^11^

The variability in non-adherence rates in studies on the BD population is attributed to several factors, including the varying definitions of adherence, different treatments, characteristics of study population, study duration and adherence assessment tools.^14^ In spite of the advance in the adherence measurement tools, they are still defective.^13^ Whilst some studies have used objective tools (such as medication serum level, urine analysis and pill count), other studies have adopted subjective measures (e.g. self- or caregiver report) or combined both types to quantify medication adherence.^15^

Non-adherence is associated with lower remission and recovery rates, higher risk of recurrence, relapse, a greater likelihood of emergency room use, hospitalisations, violence and suicides, thereby compromising the quality of life of patients and relatives, and increasing costs for the health system^9,16,17,18^ and imposing indirect economic burden.^19^

Medication adherence is a complex phenomenon that is influenced by multiple factors that could be (1) medication-related, for example, adverse effects^4^ and difficult routines^13^; (2) patient-related, such as insight, knowledge and attitude^4,13^; (3) illness-related, like presence of certain symptoms as loss of interest,^20^ or comorbidity,^21^ (4) sociocultural^22^ including stigma and social support^23^; or (5) related to the mental health service providers who frequently overlook such a problem,^4^ or offer less emotional support.^20^

Identification of potentially modifiable factors that predict treatment adherence is critical to develop effective interventions for adherence enhancement in BD populations.^13^ Very few studies, however, have specifically explored this issue amongst patients with BD.^24^

Thus, the aim of this study was to investigate the socio-demographic and clinical factors associated with medication non-adherence in Egyptian patients with BD.

## Methods

### Study design

This study was a longitudinal prospective study.

### Setting

The study was conducted over 1 year in the Institute of Psychiatry, Ain Shams University, a tertiary centre of psychiatry with a wide catchment area.

### Study population and sampling

All in-patients with BD-I who satisfied the inclusion and exclusion criteria and consented to participate were considered as the study population: retirement age in Egypt is 60 years; therefore, only adult men or women aged between 18 and 60 years, who fulfilled DSM-IV diagnostic criteria of BD-I manic episode, were included in the study. Patients with a history of any organic or severe medical co-morbidities, substance abuse or dependence, other Axis I psychiatric disorders or mental retardation were excluded.

Sample size was calculated using Epi Info Program version 6, assuming that the prevalence of BD is 1%.^25^ Approximately 40% ± 10% of patients with BD do not adhere to medications,^26^ and 95% confidence interval was considered. It was calculated to be 92 participants. However, 20% non-response rate was speculated; hence, we recruited 117 patients in the study, of whom 110 patients completed the study, whilst seven patients did not take part, with 5.9% dropout rate.

### Tools

#### Structured Clinical Interview for DSM-IV

The Arabic version^27^ to diagnose BD-I and to exclude other Axis I diagnoses.^28^

#### Young Mania Rating Scale

This is an 11-item clinician-applied scale to evaluate the severity of manic symptoms.^29^ A score of < 13 is considered normal, a score of 13–19 is considered as minimal severity, a score of 20–26 is considered as mild, a score of 27–38 is considered as moderate and that of > 38 is considered as severe. The joint reliability for total scores was 0.93, and the correlation between raters for individual items ranged from 0.66 (disruptive or aggressive behaviour) to 0.95 (sleep). The validity of the Young Mania Rating Scale (YMRS) was evaluated by comparing with other measures of mania. The correlation was 0.88 with the global measure of mania and 0.71 with the Beigel Mania Rating Scale (BMRS). The YMRS appears to be sensitive to change.^29^

#### The Clinical Global Impressions Scale

The Early Clinical Drug Evaluation Program (ECDEU) version: this is a three-item observer-rated Likert-scale measuring Illness severity (CGIS), Global improvement or change (CGIC) and therapeutic response. The Clinical Global Impression (CGI) is rated on a seven-point scale. Each component of the CGI is rated separately with no global score.^30^

#### Global Assessment of Functioning Scale

A 100-point single-item rating scale is used to indicate overall psychosocial functioning during a specified period.^31^

#### The Sheehan Disability Scale

The Sheehan Disability Scale (SDS) is a three-item brief self-report tool, whereby the patient rates the extent to which work or school, social and home life and family responsibilities are impaired by his or her symptoms on a 10-point visual analogue scale. The numerical ratings can be translated into a percentage if desired. The three items can be summed into a one-dimensional measure of global functional impairment ranging from 0 (unimpaired) to 30 (highly impaired). Functional remission was defined as SDS ≤ 6 at endpoint.^32^

#### Insight and Treatment Attitude Questionnaire

Insight and Treatment Attitude Questionnaire (ITAQ)is designed to measure the awareness of possessing mental disorder or symptoms and awareness of need for treatment by hospitalisation or by medications. It is a validated 11-item semi-structured interview that generates scores from 0 (no insight) to 22 (maximum insight). The total score is categorised into three groups: good insight (15–22), fair insight (8–14) and poor insight (0–7).^33^

#### Morisky 8-Item Medication Adherence Scale

Each item of the Morisky 8-Item Medication Adherence Scale (MMAS) measures a specific behaviour and is not a determinant of adherence behaviour. Response choices are ‘yes’ or ‘no’ for items 1–7 and a five-point Likert response for the last item. Scores range from 0 to 8, where higher scores indicate higher adherence. Scores of 8, 6 to less than 8 and less than 6 were classified as high, medium and low adherence, respectively.^34^

### Procedures

On admission patients were interviewed by the researchers using the Structured Clinical Interview for DSM-IV (SCID I) to confirm the diagnosis of BD-I. The patients were further assessed using YMRS, CGI, ITAQ, SDS and Global Assessment of Functioning (GAF) in the same setting. The second assessment was performed 6 months after discharge. YMRS, CGI, ITAQ, SDS and GAF scales were repeated by the same clinicians. Adherence was measured using MMAS. According to the rate of adherence, there were three groups of patients: high adherence group (*n* = 21), medium adherence group (*n* = 25) and low adherence group (*n* = 64).

### Statistical analysis

All data were recorded and statistical analysis was performed using the Statistical Package for Social Science (SPSS), version16.^35^ The results were tabulated, grouped and statistically analysed using the suitable statistical parameters.

Descriptive data were expressed as mean values and standard deviations. Analysis of variance (ANOVA) was used to analyse the differences amongst groups. Chi-squared test (*χ*²) was used to detect relations between categorical variables, and logistic regression analysis was used to model the relationship between adherence and other variables. For all tests, a significance level of *p* < 0.05 was pre-determined.

### Ethical considerations

Approval was obtained from the Faculty of Medicine Ethical Committee at Ain Shams University before starting the research. Informed consent was obtained from the participants and their caregivers. Participation in the study was clarified to be free, voluntary and would not imply a direct benefit for patients. Withdrawal from the study was guaranteed at any point without consequences. Confidentiality was preserved. The participants were assured that the study results would be used for scientific publication.

This article followed all ethical standards for a research without direct contact with human or animal subjects.

## Results

### Sample description

A total of 110 patients completed the study. The age range of the patients was18–60 years. There were 74 men (67.3%) and 36 women (32.7%). Of the participants, 45.5% were single, 39.1% were married, 13.6% were divorced and 1.8% were widowed. About 53.6% of participants received ≤ 12 years of education and approximately 59% were unemployed, whilst about 41% had a job. The mean duration of illness of the sample was 10.4 ± 8.7 years.

At the time of admission, YMRS mean score was as high as 37.6 ± 10.4. By CGIS, 54.5% of the sample was categorised as moderately ill, whilst almost equal portions of the sample were categorised as mildly and severally ill (20% and 20.9%, respectively). Only 1.8% were the most extremely ill.

### Prevalence of medication adherence of the study sample

**FIGURE 1:**
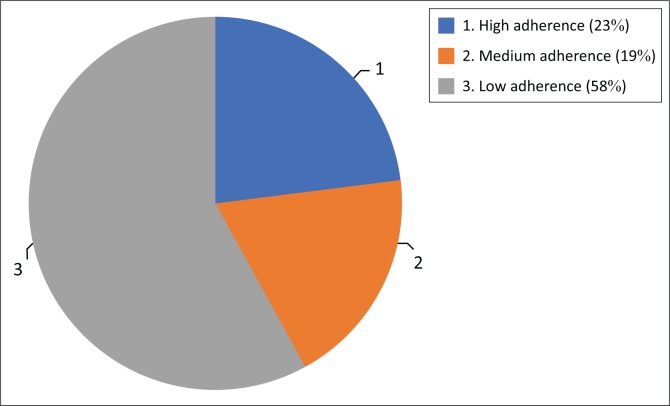
Pie chart of medication adherence assessment 6 months after discharge using Morisky 8-Item Medication Adherence Scale.

Adherence was assessed using MMAS 6 months after discharge, which revealed that 58% of patients (*n* = 64) had low adherence, whilst about one-fifth of the sample (19% – *n* = 21) were highly adherent and 23% (*n* = 25) showed medium adherence (Figure 1). Thus, we compared the three groups in terms of demographic and clinical variables.

### Comparison between the three studied groups regarding socio-demographic variables

The relationship between socio-demographic data and medication adherence in the three groups of bipolar I patients revealed that there was statistically significant difference between the three groups in terms of age (*p* = 0.005). The low adherence groups were significantly younger than the other two groups (0.007). The low adherence groups received the least years of education (9.5 ± 5.4), whilst the high adherence groups received significantly more years of education (*p* = 0.036). Most of the high adherence groups were women (76.2%), whilst those in non-adherence groups were men (*p* = 0.000). Married subjects showed higher adherence rate (71.4%) in comparison to the unmarried subjects in all groups (*p* = 0.000). Also, there was no significant difference between employment status and medication adherence (*p* = 0.9) (Table 1).

**TABLE 1a:** Comparison between the three groups regarding socio-demographic characters.

Variable	High adherent Group I (*n* = 21)	Medium adherent Group II (*n* = 25)	Low adherent Group III (*n* = 64)	ANOVA		Post hoc test		
				*f*	*p*	G1 versus G2	G2 versus G3	G1 versus G3
Age (mean ± s.d.)	40.2 ± 10.7	37.2 ± 9.7	32.5 ± 9.6	5.6	0.005*	0.105	0.571	0.007*
Educational years (mean ± s.d.)	12.7 ± 3.8	11.48 ± 5.4	9.5 ± 5.04	3.663	0.029*	0.023*	0.696	0.036*

ANOVA, analysis of variance; s.d., standard deviation.

* , *p* < 0.05 statistically significant.

**TABLE 1b:** Comparison between the three groups regarding socio-demographic characters.

Variables	Group I		Group II		Group III		Chi-square	
	*n*	%	*n*	%	*n*	%	*X*^2^	*p*
**Gender**							23.7	0.000**
Male	5	23.8	17	68	52	81.3	**-**	-
Female	16	76.2	8	32	12	18.8	**-**	-
**Marital status**							24.1	0.000**
Single	1	4.8	10	40	39	60.9	**-**	**-**
Married	15	71.4	10	40	18	28.1	**-**	**-**
Divorced	5	23.8	5	20	5	7.8	**-**	**-**
Widow	0	0	0	0	2	3.1	**-**	**-**
**Employment**							0.04	0.9
Unemployed	12	57.1	15	60	38	59.4	**-**	**-**
Employed	9	43.9	10	40	26	40.6	**-**	**-**

** , *p* < 0.001 statistically highly significant.

### Comparison between the three studied groups in terms of clinical variables

Although there was no statistically significant difference between medication adherence and the number of episodes, the number of previous hospital admissions and the duration of index episode yet, Group I showed the longest duration of illness (13.8 ± 11.2) years and Group III demonstrated the shortest duration of illness (*p* = 0.009) (Table 2).

**TABLE 2a:** Comparison between groups regarding clinical characteristics.

Variable	High adherence Group I (*n* = 21)	Medium adherence Group II (*n* = 25)	Low adherence Group III (*n* = 64)	ANOVA	
				*f*	*p*
Duration of illness (M ± s.d.)	13.80 ± 11.20	13.08 ± 9.00	8.30 ± 8.70	4.800	0.009*
Number of episodes (M ± s.d.)	4.40 ± 2.20	7.60 ± 6.40	5.70 ± 5.20	2.230	0.100
Number of admission (M ± s.d.)	2.09 ± 2.09	4.04 ± 3.40	3.09 ± 2.70	2.750	0.060
Duration of index episode	29.50 ± 33.70	34.60 ± 32.30	34.60 ± 25.20	0.300	0.700
YMRS on admission	30.50 ± 11.70	37.70 ± 8.07	39.90 ± 9.90	6.978	0.001*
YMRS 6 months later	2.10 ± 2.40	6.50 ± 6.00	16.30 ± 13.80	16.297	0.000**

ANOVA, analysis of variance; s.d., standard deviation; M, mean.

* , *p* < 0.05 statistically significant;

** , *p* < 0.001 statistically highly significant.

**TABLE 2b:** Comparison between groups regarding clinical characteristics.

Variable	Group I		Group II		Group III		Chi-square	
	*n*	%	*n*	%	*n*	%	*X*^2^	*p*
**ITAQ on admission**							26.8	0.000**
Good	9	42.9	1	4	9	14.1	-	-
Fair	0	0	9	36	5	7.8	-	-
Poor	12	57.1	15	60	50	78.1	-	-
**ITAQ 6 months later**							50.9	0.000**
Good	20	95.2	17	68	10	15.6	-	-
Fair	1	4.8	3	12	10	15.6	-	-
Poor	0	0	5	20	44	68.8	-	-
**YMRS on admission**							19.5	0.003*
Minimal	5	23.8	0	0	2	3.1	-	-
Mild	4	19.0	3	12	4	6.3	-	-
Moderate	8	38.1	10	40	24	37.5	-	-
Severe	4	19.0	12	48	34	53.1	-	-
**YMRS 6 months later**							25.5	0.001*
Normal	21	100.0	23	92	34	53.1	-	-
Minimal	0	0	0	0	10	15.6	-	-
Mild	0	0	2	8	8	12.5	-	-
Moderate	0	0	0	0	6	9.4	-	-
Severe	0	0	0	0	6	9.4	-	-
**CGI on admission**							23.4	0.003*
Mild	3	14.3	0	0	0	0	-	-
Moderately ill	2	9.5	5	20	15	23.4	-	-
Markedly ill	14	66.7	12	48	34	53.1	-	-
Severely ill	2	9.5	6	24	15	23.4	-	-
Most extreme	0	0	2	8	0	0	-	-
**CGI 6 months later**							32.6	0.000**
Normal	13	61.9	8	32	7	10.9	-	-
Borderline	8	38.1	13	52	26	40.6	-	-
Mild	0	0	2	8	13	20.3	-	-
Moderate	0	0	2	8	9	14.1	-	-
Markedly	0	0	0	0	5	7.8	-	-
Severely	0	0	0	0	4	6.3	-	-

ITAQ, Insight and Treatment Attitude Questionnaire; YMRS, Young Mania Rating Scale; CGI, Clinical Global Impression.

* , *p* < 0.05 statistically significant;

** , *p* < 0.001 statistically highly significant.

Insight to illness as measured by ITAQ is presented in Table 2. Data reveal a significantly better insight amongst high adherence group on admission (42.9%) and 6 months after discharge (95.2%), whilst most of the low adherence patients lacked insight either on admission (78.1%) or 6 months after discharge (68.8%) (*p* = 0.000). A strong inverse association was found in relation with severity of illness as indicated by YMRS and SCGI scores both on admission (*p* = 0.003) and after 6 months of discharge (*p* ≤ 0.001) (Table 2).

### Relation between medication adherence and patient’s functioning

Data showed that the high adherence groups had the highest mean score of GAF on admission (40.5 ± 13.2) and 6 months after discharge (82.5 ± 7.8) compared to the other groups (*p* = 0.003 and *p* = 0.000 correspondingly).

In the meantime, the scores of SDS on admission did not show any significant differences amongst the three groups. Whilst at the 6-month follow-up, the high adherence group scored the lowest disability score (4.1 ± 3.1). On the other hand, the low adherence group scored the highest score in disability (Table 3).

**TABLE 3:** Medication adherence in relation to clinical improvement and functioning.

GAF	MMAS			ANOVA	
	High adherence Group I (*n* = 21)	Medium adherenceGroup II (*n* = 25)	Low adherenceGroup II (*n* = 64)	*F*	*p*
On admission	40.5 ± 13.5	27.9 ± 14.50	28.7 ± 13.9	6.234	0.003*
6 months later	82.5 ± 7.8	69.8 ± 9.08	59.03 ± 18.4	20.044	0.000**
**She ehan Disability Scale**					
On admission	20.1 ± 6.6	21.8 ± 6.00	22.2 ± 6.5	0.814	0.446
6 months later	4.1 ± 3.1	7.4 ± 3.70	13.7 ± 8.0	20.465	0.000**

GAF, Global Assessment of Functioning; MMAS, Morisky 8-Item Medication Adherence Scale; ANOVA, analysis of variance.

* , *p* < 0.05 statistically significant;

** , *p* < 0.001 statistically highly significant.

### Predictive factors related with adherence to medications

To evaluate the predictive value for the previously analysed factors, we used logistic regression analysis tests. We used score 6 as the cut-off point for MMAS, as patients who scored ≥ 6 were categorised as adherent and the patient who scored < 6 were categorised as non-adherent. Adherence assessment 6 months after discharge was used a dependent factor, and then we used variables that showed statistically significant relations (Table 4).

**TABLE 4:** Logistic regression analysis for the potential predictive factors for adherence.

Factors	*B*	s.e.	Wald	Sig.	Exp (B)
Age	−0.102	0.042	5.784	0.016*	0.903
Male gender	−0.304	0.792	0.148	0.701	0.783
Years of education	−0.005	0.071	0.005	0.943	0.995
GAF 6 months after discharge	0.034	0.050	0.468	0.494	1.035
Insight 6 months after discharge	4.110	1.223	11.294	0.001*	60.953
YMRS 6 months after discharge	0.006	0.043	0.019	0.889	1.006

GAF, Global Assessment of Functioning; YMRS, Young Mania Rating Scale; s.e., standard error.

* , *p* < 0.05 statistically significant.

Despite that there are a number of significant differences in some variables in the univariate analysis, they did not show any predictive factors in the regression analysis. Indeed, amongst all analysed factors, only age (*p* = 0.016) and insight (*p* = 0.2) were considered potential predictive factors for medication adherence in our study (Table 4).

## Discussion

Poor adherence to treatment is one of the main challenges for controlling the symptoms and preventing the recurrence in BD. Despite the magnitude of functional losses and disability worldwide amongst people with BD, non-adherence is a continuing and frequent phenomenon, associated with severe clinical consequences, and reduces the quality of life of patients.^32^

In the current study, the majority (58%) of patients show low adherence rate to their medical regimens. Comparable to our results, many studies have reported the incidence of non-adherence amongst bipolar populations ranging from 20% to 70%.^7,8,9,10^ The wide variation in the rate of non-compliance can be explained by the use of different modalities for assessment, which, although becoming more sophisticated, are still unable to produce precise results.^32,36^ In the current study, MMAS was used because of feasibility reasons, including unavailability of electronic packages and impracticality of applying measures based on medication acquisition or possession because of difficulties in monitoring dispensed prescriptions in Egypt. Direct measurement of levels of drugs was inapplicable in the current study as patients were prescribed various mood stabilisers.

Several socio-demographic and clinical factors are proposed to be related to non-adherence. Hence, the focus of this current research is on further study of these factors.

### Socio-demographic factors

Regarding age, the results of our study were consistent with previous studies,^37,38,39^ in that younger age might be a risk factor for non-adherence to medication. This could be explained by a better understanding of the nature of the illness, its course and experience with treatment and hospitalisation in older patients.^15^ On the contrary, one study reported better compliance with younger patients.^40^ Moreover, several researches revealed no impact of age on the pattern of adherence.^41,42^

Consistent with previous studies, our results showed that female patients were more adherent to medication than male patients.^43^ In contrast, one study found that female patients have poorer adherence than male patients,^37^ whilst other studies reported no significant relation or did not confirm such findings.^40,42^ Social and cultural factors may explain this disparity.

Our findings were in line with previous research,^43,44,45^ which highlighted that marriage is positively associated with adherence to medication, emphasising the role of social support in motivating the patients to adhere to treatment. However, some researchers have found either a significant difference in adherence based on marital status^46^ or equivocal results.^42^ Furthermore, high adherence was associated with higher educational level and this is similar to the findings of some studies.^47^ This may reflect the importance of education in better understanding of the symptoms and illness, although this has not been the case for every study.^42^

### Clinical characteristics

The literature is non-conclusive regarding the relationship between different clinical factors in BD (such as the number and frequency of episodes, hospitalisation, type and severity of symptoms, duration of illness and characteristics of index episode) and medication adherence.^8^

This study, in agreement with previous studies,^44,46,48^ found a positive association between the duration of illness and adherence, as individuals seem to acquire better adherence over time.^46^ In contrast, one study^49^ found that the longer duration of disease was associated with greater compliance. Other studies, however, found no significant relationship between the duration of illness and adherence.^41,50^

Our results identified no significant relationship between the number of episodes and adherence, in line with previous literature.^46,50^ Conversely, some studies showed that high episode frequency is associated with worse medication adherence.^43,51^ This was explained by progressive decline in function and cognitive functions associated with recurrent episodes and that better medication adherence may be reflected by decreasing number of relapse and recurrence for the adherent patients. Other data suggest that lower number of episodes is related to non-adherence.^9^

Interestingly, some studies found more hospital admissions in adherent patients,^29,43^ whereas others^50,48^ observed the converse. In our study, the number of hospital admissions was not found to be related to medication adherence, which is in agreement with several previous researches.^41,45^ Likewise, the present study found that there is no statistically significant association between the duration of index episode and medication adherence.

Our data revealed that the severely ill patients, as assessed by YMRS and CGI, demonstrated a low rate of adherence. That is probably because of their low ability to engage in the treatment process and poor judgement.^52^ Similarly, the literature indicates consistently that more severe BD symptoms are associated with worse medication adherence.^13,52,53^ In the same context, previous studies found that episodes with high score on CGI scale are more at risk of non-adherence to medication.^45^ On the contrary, other studies did not detect any effect of severity of symptoms on the pattern of adherence.^41^

We investigated the influence of insight and illness severity on the adherence rate. Impaired insight is regarded as an important feature of bipolar patients that contributes to functional outcome, prognosis and treatment adherence. The low adherence group in our results showed high rate of poor insight on admission and after 6 months, which is consistent with previous studies,^11,15,45^ where the patients’ denial of their need for treatment was the most often patient-cited reason for non-adherence. Similarly, researchers^13,54^ found that non-adherence was linked to denial of illness severity and denial of therapeutic effectiveness. Additionally, it was found^45^ that good insight seems to be a protective factor for good adherence.

### Functioning of the patients

Studying the relationship between adherence and functioning was done by GAF scale and SDS. Our results showed a statistically significant positive association between better adherence and good functioning and less disability 6 months after discharge. These results are inconsistent with the literature, which indicates that poorly adherent patients showed worse functioning compared with those with high levels of adherence.^53,55^ In their follow-up study, Novick et al.^56^ identified a positive association between functioning and treatment adherence at baseline, in agreement with our results, yet a slightly negative association on follow-up after a year of treatment of bipolar patients.

On assessing disability in bipolar patients during remission phase, a significant association was not found between treatment adherence and disability,^57^ which contrasts with our results. The results could be explained by bidirectional effect of both adherence and functioning, as better level of functioning on initiation of treatment would improve adherence, and then good treatment adherence would positively impact functional outcome, thereby reducing the level of disability as the therapy continued. Despite the growing body of research on adherence in treatment and its determinants, association studies with level of functioning and disability at different phases of bipolar remain limited, inviting further exploration in this domain.

### Predictive factors

Using logistic regression analysis of the different variables that were statistically significant related to adherence, the independent predictors of medication adherence included two factors: age and insight 6 month after discharge. Our results are in agreement with the previous studies.^24,38,39,50,51^

Despite the association found between gender, marital status, years of education, GAF and severity of illness 6 months after discharge and medication adherence on univariate analysis, multivariate statistics did not reveal a predictive correlation between the named variables, which agrees with the results of a previous study.^58^ On the other hand, some studies identified other predictive values, such as female gender ^38^ and severity of illness.^51^

## Strength and limitations

Although our study is one of the preliminary studies in Egypt interested in exploring factors affecting adherence to medication amongst patients with BD-I, an important limitation of this study is its reliance on self-reporting. Objective assessments could have decreased any variance because of error. Another limitation lies in the difficulty in generalising the data because of the small sample size and selection of patients with BD-I (manic episode only). Thus, our findings may not be generalised to other BD subtypes or BD-I patients in other mood states. Therefore, more research should be performed on larger samples. Cultural concepts of mental illness in Egypt were not in the scope of the current study, which would have provided a better understanding of causes of non-adherence in our sample.

### Implications

Our results draw attention to the amplitude of non-adherence and its relationship with functioning in a sample of patients with BP-1, highlighting the need to augment treatment adherence to reduce the disability in such patients. Psychosocial interventions have been applied in order to improve adherence^7^ by employing cognitive behavioural therapy (CBT),^14^ motivational interviewing^59^ and psychoeducation^14,59^ principles. More focused therapies were coined such as customised adherence enhancement (CAE)^60^ and improving treatment adherence programme^21^ to address this challenge. Whilst therapies focusing on patients and their risk factors might be promising in improving adherence,^7^ some studies have shown the involvement of families and carers to be of added benefit.^14,21^

## Conclusion

Several socio-demographic and clinical variables were found to be associated with low adherence rate to the prescribed medication in patients with BD-I. Age and impaired insight were found to be significant predictive factors for non-adherence.
